# Supplementary material for: Spatially Resolved Correlation between Stiffness Increase and Actin Aggregation around Nanofibers Internalized in Living Macrophages
Source: Materials (Basel). 2020 Jul 21;13(14):3235. doi: 10.3390/ma13143235 (PMC7412258; doi:10.3390/ma13143235)
Supplement: Supplementary file 1 [file materials-13-03235-s001.pdf]

materials

MDPI

Supplementary Information

Spatially Resolved Correlation between Stiffness Increase and Actin Aggregation around Nanofibers Internalized in Living Macrophages

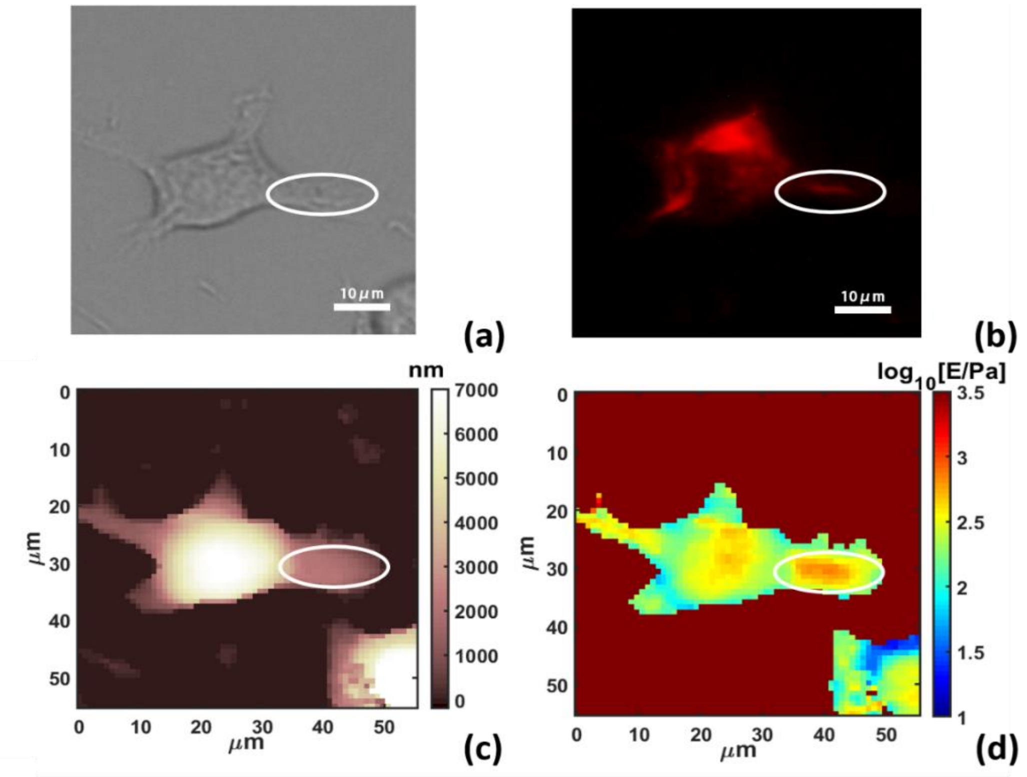

Figure S1. Optical microscopy and AFM colocalized observation of the cell presented in Figure 3 of main text. (**a**) optical transmission image, (**b**) fluorescence image, (**c**) AFM height image, (**d**) Young's Modulus mapping.

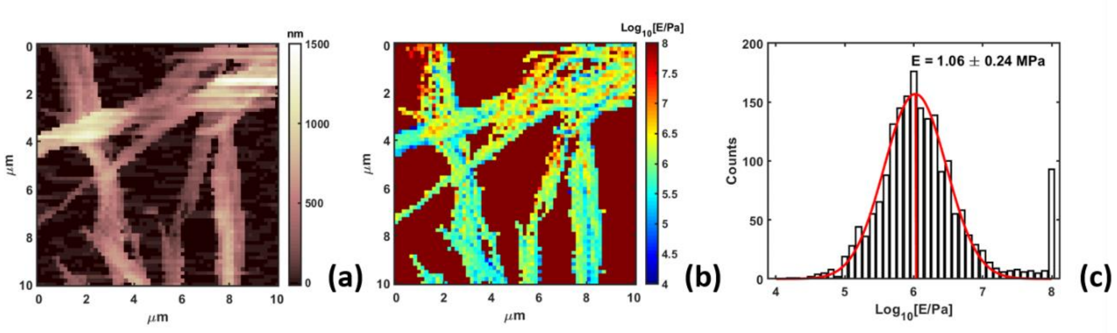

Figure S2. Nanomechanical analysis in PBS with AFM for PLGA-PEG nanofibers used in this study after deposition on mica. (**a**) Morphology, (**b**) Young's Modulus map, (**c**) quantification histogram of Young's Modulus.

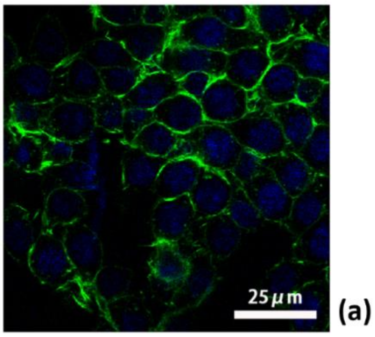

Figure S3. CLSM images of fixed control. No red emission is detected due to absence of nanofibers in culture medium. (Blue: DAPI for nuclei, green: Phalloidin for actin), (**a**) low magnification, scale bar 25 μm, (**b**) high magnification, scale bar 7.5 μm.

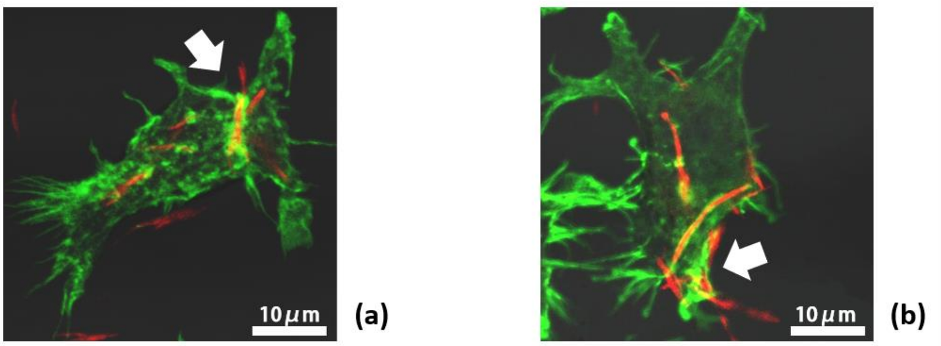

Figure S4. CLSM images of fixed cells showing the internalized PLGA-PEG nanofibers after 14h and actin distribution (Scale bar: 10 μm. Blue: DAPI for nuclei, green: Phalloidin for actin, red: Nile Red for nanofibers, white arrow shows actin wrap around nanofiber), (**a**,**b**) high magnification scale bar 10 μm.

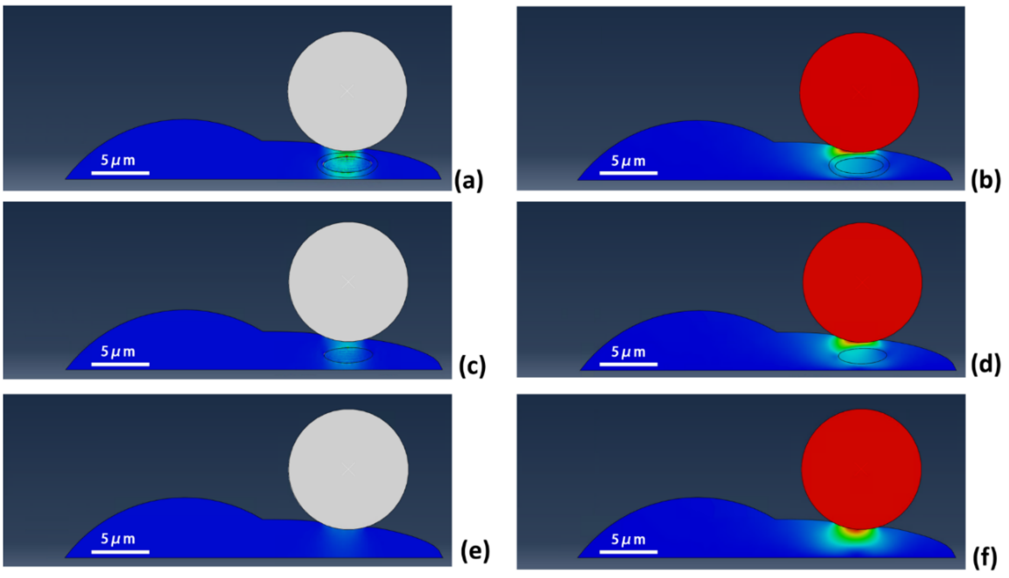

Figure S5. Normal stress field and magnitude of normal displacement field for FEM simulation of (a) and (b) PLGA-PEG nanofiber with 500 nm actin wrap, (c) and (d) PLGA-PEG nanofiber without wrap, e) and (f) cell body without heterogeneity. Simulation fields were fixed at 600 nm of indentation after contact point.
